# Supplementary material for: Flavonoid Synthesis by Deinococcus sp. 43 Isolated from the Ginkgo Rhizosphere
Source: Microorganisms. 2023 Jul 21;11(7):1848. doi: 10.3390/microorganisms11071848 (PMC10386165; doi:10.3390/microorganisms11071848)
Supplement: Supplementary file 1 [file microorganisms-11-01848-s001.zip › microorganisms-2453780-supplementary.pdf]

1. The numbers at the nodes indicate the levels of bootstrap support based on Kimura distance and the neighbour-joining method.

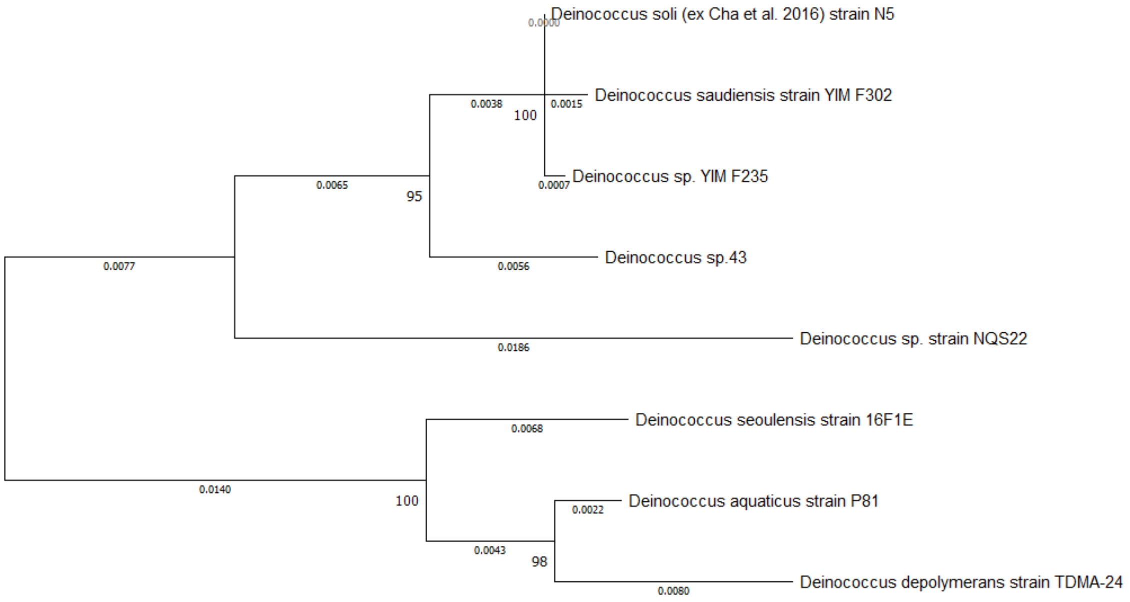

Figure S1. The numbers at the nodes indicate the levels of bootstrap support based on Kimura distance and the neighbor-joining method.

2. Homologous evolutionary relationship of key genes in the upstream flavonoid synthesis pathway. Evolutionary analysis was performed using MEGA X. Clustal W was used to compare each protein sequence. Based on the Poisson model, the phylogenetic tree was constructed by NJ (Neighbor-joining) method and evaluated with bootstrap value 5000. In order to further confirm the Protein homology, the similarity search of Hidden Markov model (HMM) (E value <1e-5) was used to identify the characteristic Protein family (Pfam) domains contained in the two closest phylogenetic protein sequences. Relevant protein sequences were submitted to InterProScan 5 for conserved domain search.

(1).CHI:

> KALODDLK_00111 tRNA 5-methylaminomethyl-2-thiouridine biosynthesis bifunctional protein MnmC

MIGAGIAGASVAYFLGRAGAQVTVVDAGAHRASDVPSALINPVRGQSGGVDAR-
ALDGMRFT-
WTLLRDLKAAGHAVPHAQLGVLRPIPDDRARARFERNLPAALNHAWLSSADAPEPL
APGWGHVLHLPDGGWVDGPAFTRALVQASGAQVVTGRAQDW-
TARTVTLAGGDTLSGDAV-
VFCGGSVGVTWRGEAATHRRGTLLTLDRAVTRVPLSFGAYLAPDARGGVLGATFETPS
PTWTPDGLPLASLGWLLGKGAALTDLRGARVTGHWTGTRLSGLNAG-
PQGGGTWWLTGLSSKGFLLGPLLAAELASELMSAARPG

> WP_029242752.1 FAD-dependent oxidoreductase [Pseudomonas viridiflava]

MSTLFETDALIIGGGIVGASAALALALKGKRVALLERDFCGSHSSGVNY-
GGVRRQGRPLSQLPLSQRAHHIWGNLRDLIGIDGEYQRSGHLKLARSDQDMYALRAY
AQASQGFGLDLQLLDRAELRARF-
PWAGDVAVGASLCADDGHANPRLVSPAFARAAR-
LAGAQVFEQAQVTQVNHDGQAFIVETANGLKLRAPWLLNCAGAWAGQLAAQFNE
PVPMYSGHPAMLVTEPLPMFMDVSTGVEGGGIYARQVARGNCVLGGGQGFALD-
PAR-
TRPGQAAVLDILRNAVELYPPLKGAQAIRTWSGTEGYLPDREPVLGPSLAQPGLLHGF

GFAGAGFQIGPAAGEALAEWVCAGHSSISLDAFSIGRFRTEQALSITDSTLIVSPRSA-PEPVSNVIPLHRGRSPL

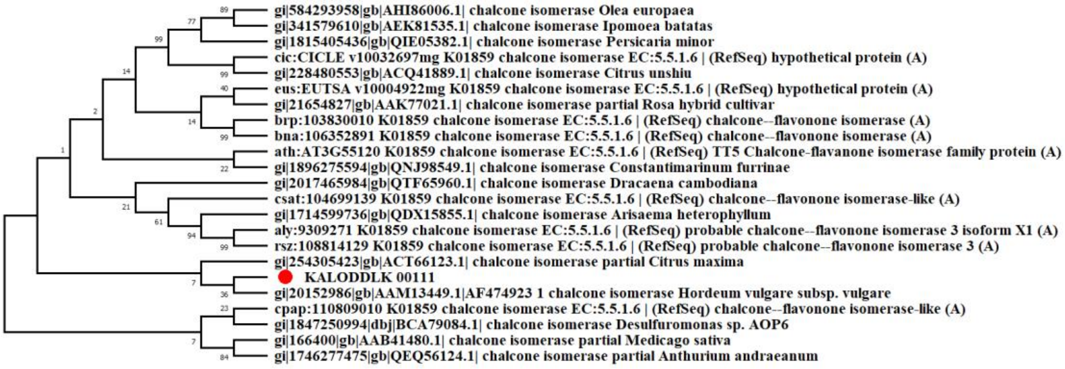


```
549                           MIGAGIAGASVAYFLGRAGAQVTVVDAGAHRASDVPSALINPVRGQSGGVDARALDGMRF 60
gi|1847250994|dbj|BCA79084.1| MIV--------RLLL--------------------SLLLLAPLPVL-----AVEVEGVQL 27
                              **           :*                       *: *:        *  ::*:::

549                           TWTLLRDLKAAGHAVPHAQLGVLRPIPDDRARARFERNLPAALNHAWLSSADAPEPL--- 117
gi|1847250994|dbj|BCA79084.1| ----PDRLEVQGETLHHNGHGIRKKLF-------FD---------IYIGSLYTATPVRSL 67
                                     *:. *.:: *   *: : :        *:          ::.*  :  *:

549                           -----APGWGHVLHLPDGGW--VDG----PAFTRALVQASGAQVVTG----RAQDWTART 162
gi|1847250994|dbj|BCA79084.1| SETLASPGAKLI--LMDFLYKKVEKEKIIEAFREGLANQSAA-LASGEEAKAFLAWF--- 121
                                   :**   :  * *  :  *:      ** ..*.: *.* :.:*        *

549                           VTLAGGDTLSGDAVVFCGGSVGVTWRGEAATHRRGTLLTLDRAVTRVPLSFGAYLAPDAR 222
gi|1847250994|dbj|BCA79084.1| ----GRDFVRGDRVELELGGDG-----TVTARHNGELLGTLHSTAFAEAVLGIYLGDK-- 170
                                  * * : ** * :  *.  *      .::::.* **   ::.: .   :* **. .

549                           GGVLGATFETPSPTWTPDGLPLASLGWLLGKGAALTDLRGARVTGHWTGTRLSGLNAGPQ 282
gi|1847250994|dbj|BCA79084.1| --------------------------------PADARLKAGMLQGKER------------ 186
                                                               * : *:.. : *:

549                           GGGTWWLTGLSSKGFLLGPLLAAELASELMSAARPG 318
gi|1847250994|dbj|BCA79084.1| ------------------------------------ 186
```

(2).CHS:

>KALODDLK_00431 1,3,6,8-tetrahydroxynaphthalene synthase

MRRMPLTPHLRALVTGTPPHLTPQTQVQEAARTLFPRMAARPQLLDVFT-NAMIDTRALARPLEWYLTPRGFGEKNAVFVQEARALTRRLAQEALAAAQITPADVDAVVVVNTSGISAPSLDADLIEHLGINRHAARLPVWGLGCAGGASGLARAADLVRA-GYR-RVLYVAVELCSLTLVHGDETKSNFVGTALFSDGGAAAVLTHPDEPGPAPLAELCGAYSTLIEDSEDIMGWDVVDEGLKVRFSRDIPTLVRG-MMQGNVQAALSAHGWCPTQIGTYVVHPGGVKVLSAYEEALNLPAGALDASRHVLRHYGNMSSVTVLFVLQETLRAHPQGRALLSAMGPGFSAEHVLLNFP

>CNM35526.1 chalcone synthase [Mycobacterium tuberculosis]

PPHLTPQTQVQEAARTLFPRMAARPQLLDVFTNAMIDTRALARPLEWYLT-PRGFGEKNAVFVQEARALTRRLAGEALDAAQITPANVDAVVVVNTSGISAPSLDADLIEHLGINRHAARLPVWGLGCAGGASGLARAADLVRAGYRRVLYVAVELCSLT-LVHGDETK-SNFVGTALFSDGGAAAVLTHPDEPGPAPLAELCGAYSTLIENSEDIMGWDVVDEGLKVRFSRDIPTLVRGMMQGNVQAALSAHGWCPTQVGTYVVHPGGVKVLSAYEEAL-NLPAGAL-DASRHVLRHYGNMSSVTVLFVLEETLRAHPQGRALLTAMGPGFSAEHVLLNF

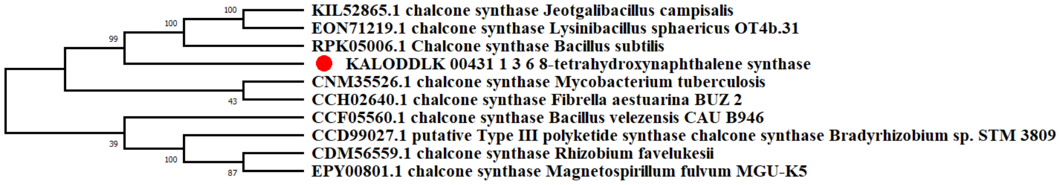


```
KALODDLK_00431    MRRMPLTPHLRALVTGTPPHLTPQTQVQEAARTLFPRMAARPQLLDVFTNAMIDTRALAR    60
CNM35526.1        -----------------PPHLTPQTQVQEAARTLFPRMAARPQLLDVFTNAMIDTRALAR    43
                                   *******************************************

KALODDLK_00431    PLEWYLTPRGFGEKNAVFVQEARALTRRLAQEALAAAQITPADVDAVVVVNTSGISAPSL    120
CNM35526.1        PLEWYLTPRGFGEKNAVFVQEARALTRRLAGEALDAAQITPANVDAVVVVNTSGISAPSL    103
                  ****************************** *** *******:*****************

KALODDLK_00431    DADLIEHLGINRHAARLPVWGLGCAGGASGLARAADLVRAGYRRVLYVAVELCSLTLVHG    180
CNM35526.1        DADLIEHLGINRHAARLPVWGLGCAGGASGLARAADLVRAGYRRVLYVAVELCSLTLVHG    163
                  ************************************************************

KALODDLK_00431    DETKSNFVGTALFSDGGAAAVLTHPDEPGPAPLAELCGAYSTLIEDSEDIMGWDVVDEGL    240
CNM35526.1        DETKSNFVGTALFSDGGAAAVLTHPDEPGPAPLAELCGAYSTLIENSEDIMGWDVVDEGL    223
                  *********************************************:**************

KALODDLK_00431    KVRFSRDIPTLVRGMMQGNVQAALSAHGWCPTQIGTYVVHPGGVKVLSAYEEALNLPAGA    300
CNM35526.1        KVRFSRDIPTLVRGMMQGNVQAALSAHGWCPTQVGTYVVHPGGVKVLSAYEEALNLPAGA    283
                  *********************************:**************************

KALODDLK_00431    LDASRHVLRHYGNMSSVTVLFVLQETLRAHPQGRALLSAMGPGFSAEHVLLNFP    354
CNM35526.1        LDASRHVLRHYGNMSSVTVLFVLEETLRAHPQGRALLTAMGPGFSAEHVLLNF-    336
                  ***********************:*************:***************
```

(3).F3H:

>KALODDLK_02511 Catechol-2,3-dioxygenase

MTTDSVLPASTHVGAVHLNVRDLNGTAAFYATLLGLSATTLTETDVTLAAHGT-PLLHL-HAAPDLPRAPVSRPGLYHTAFLLPTRAALGRWLAHAARLGHRIGSGDHLVSEAFYLNDPEGNGIEVYADRPRDTWTWRDGQVQMDTKAVDAAAVLASAGIDPATLDGTAP-FTAPQGTTVGHVHLKVGSAAQAARWYADTLGLDVVADLGSAAFLSWGGYHHHVGLNEWHSAGQPAPSTPAAGLAGVTFHAPDLGALRAHLAGRADVQNEGGHLTLRD-PWGNRVTVAQ

>svs:117847667 K00475 naringenin 3-dioxygenase [EC:1.14.11.9] | (RefSeq) flavanone 3-dioxygenase 1 isoform X1 (A)

MLLYKAAAQAAYGVRYEQQPSAFRSTIIDREAAARTTTTMAPASSAAPFLPT-TASSEAT-LPPSFVREEDERPKVPHDSFSDEVPVISLDGIGGGERRAEIRARVAAACEDWGIFQVVDHGVDAALVAEMARLARDFFALPPQEKLRFDMSGGKKGG-FIVSSHLQGEAVQDWREIVTY-FSYPVKARDYSRWPDKPAAWRAVVEQYSEQLVGLSCKLLGVLSEAMGLEAGALAEACVEMDQKVVVNFYPRCQQPDLTLGLKRHTDPGTITLLLQDLVG-GLQATRDGGRTWITVQPVEGAFVVNLGDHGHFLSNGRFKNADHQAVVNSECSRLSIATFQNPAPDATVYPLAVREGEAPILDEPITFAEMYRRKMARDIE-VAKLKKQARAAAEKQQVQVQMPQQSASEEFAVPKPESLDEILA

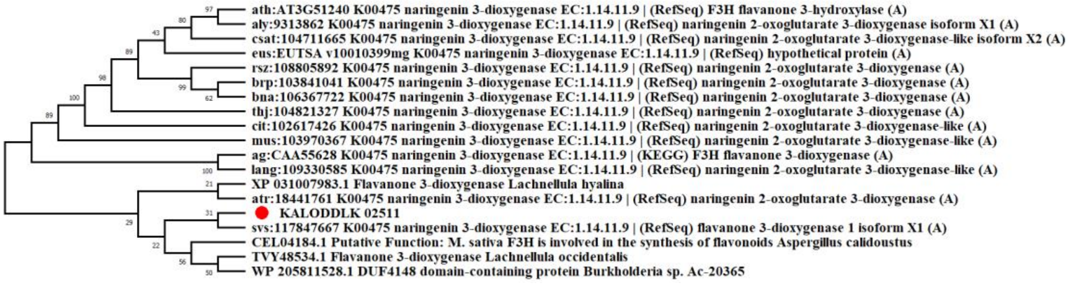

```
KALODDLK_02511     MTTDSVLPASTHVGAVHLNVRDLNGTAAFYATLLGLSATTLTETDVTLAAHGTPLLHLHA     60
svs:117847667      ----MLLY--KAAAQAAYGVRYEQQPSAFRSTIIDREAAARTTTTMAPASSAAPFLPTTA     54
                       :*   . .. .  .**  :  :** :*::. .*:: * * :: *: .:*:*   *

KALODDLK_02511     ------APDLPRAPVSRPGLYHTA------------------------------------     78
svs:117847667      SSEATLPPSFVREEDERPKVPHDSFSDEVPVISLDGIGGGERRAEIRARVAAACEDWGIF     114
                         *.: *   .** : * :

KALODDLK_02511     ----------------------FLLPTRAALG---------RWLAHAARLGHRIGSGDHL     107
svs:117847667      QVVDHGVDAALVAEMARLARDFFALPPQEKLRFDMSGGKKGGFIVSSHLQGEAVQDWREI     174
                                         * ** :  *           ::. :   *. : .  .:

KALODDLK_02511     VSEAFYLNDPEGNGIEVYADRPRDTWTWRDGQVQMDTKAVDAAAVLA-----SAGIDPAT     162
svs:117847667      V---TYFSYPV--KARDYSRWPDKPAAWRAVVEQYSEQLVGLSCKLLGVLSEAMGLEAGA     229
                   *   *:. *    . *:  * .  :**   * . : *. :. *      : *:: .:

KALODDLK_02511     LDGTAPFTAPQGTTVGHVHLKVGSAAQAARWYAD------TLGLDVVADLGSAAFL----     212
svs:117847667      LAEAC--------------VEMDQ-KVVVNFYPRCQQPDLTLGLKRHTDPGTITLLLQDL     274
                   *  :.              ::::..   ...:*        ****.  :* *: ::*

KALODDLK_02511     --------------------------SWGGYHHHVGLNEWHSAGQPA-PSTPAAGLAGVT     245
svs:117847667      VGGLQATRDGGRTWITVQPVEGAFVVNLGDHGHFLSNGRFKNADHQAVVNSECSRLSIAT     334
                                             . *.: *.:. ..::.*.: *  .: .: *: .*

KALODDLK_02511     FHAPDLGALRAHLAGRADVQNEGGHLTLRDPWGNRVTVAQ    285
svs:117847667      FQNPAPDATVYPLA-----VREGEAPILDEP---------    360
                   *: *  .*    **      .**    * :*
```

(4).4CL:

>KALODDLK_02763 30S ribosomal protein S12

VCTRVYTTTPKKPNSALRKVCRVRLTNGFEVTSYIGGEGHNLQEHSVILIRG-GRVKDLPGVRYHTVRGALDCSGVKDRKQARSKYGVKRPKA

>BAE77949.1 30S ribosomal subunit protein S12 [Escherichia coli str. K-12 substr. W3110]

MCTVVKTTTPKKPNSALRKIARVRLSSAFEVTAYIPGEGHNLQEHSVVLIRG-GRVKDLPGVRYHIVRGSLDTQGVKDRNKSRSKYGTKKPKA

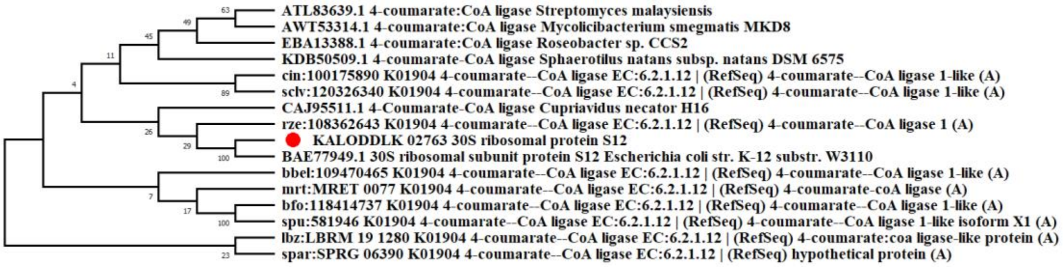


```
KALODDLK_02763     VCTRVYTTTPKKPNSALRKVCRVRLTNGFEVTSYIGGEGHNLQEHSVILIRGGRVKDLPG     60
BAE77949.1         MCTVVKTTTPKKPNSALRKIARVRLSSAFEVTAYIPGEGHNLQEHSVVLIRGGRVKDLPG     60
                   :** * *************:.****:..****:** ***********:************

KALODDLK_02763     VRYHTVRGALDCSGVKDRKQARSKYGVKRPKA     92
BAE77949.1         VRYHIVRGSLDTQGVKDRNKSRSKYGTKKPKA     92
                   **** ***:** .*****:::*****.*:***
```

(5).C4H:

>KALODDLK_01569 Putative cytochrome P450 139

GYDIPAESKILVNAWFLANDPKQWVRPDEFRPERFLGEEKAV-KADGNDFRFVPFGVGRRSCPGIILALPIIGITLGRL

> ACE88962.1 cinnamic acid 4-hydroxylase, partial [Phyllostachys edulis]

GVRLPRGALALYSPYLSGRDPAVWDRPDEFDPGRWAGKPPAWAYLPFGG-GERLCLGMHLAQMLIHDALAAL

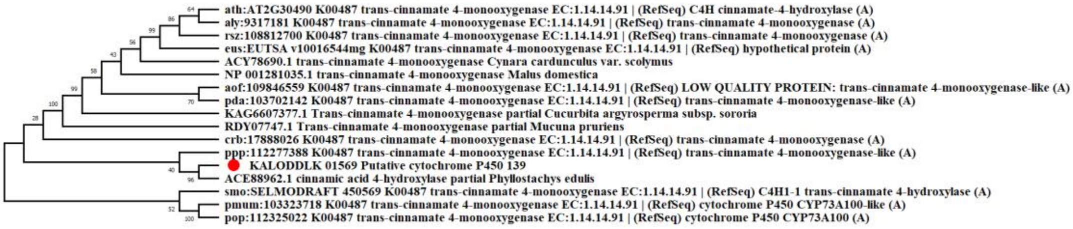


```
KALODDLK_01569      GYDIPAESKILVNAWFLANDPKQWVRPDEFRPERFLGEEKAVKADGNDFRFVPFGVGRRS      60
ACE88962.1          GVRLPRGALALYSPYLSGRDPAVWDRPDEFDPGRWAGKPP-------AWAYLPFGGGERL      53
                    *   :*   :   * .  :: ..**   * ***** * *: *:          :  ::*** *.*

KALODDLK_01569      CPGIILALPIIGITLGRL   78
ACE88962.1          CLGMHLAQMLIHDALAAL   71
                    * *: **   :*   :*.  *
```

(6).F3′5′H(CYP75A):

>KALODDLK_01569 Putative cytochrome P450 139

AVMKEVLRLHPILPLMVPHCPSESCVVGGYTIPKGARVFVNVWAIHRDPSI-WENPTEFDPERFVDGKGDYSGKDFTYFPFGSGRRICAGIAMAERMVMFSLASL

> PSS11496.1 Flavonoid 3',5'-hydroxylase [Actinidia chinensis var. chinensis]

AALKEVLRLYPPGWMGSRRLSRDLIWGGVRLPRGALALYSPYLSGRD-PAVWDRPDEFDPGRWAGKPPAWAYLPFGGGERLCLGMHLAQMLIHDALAAL

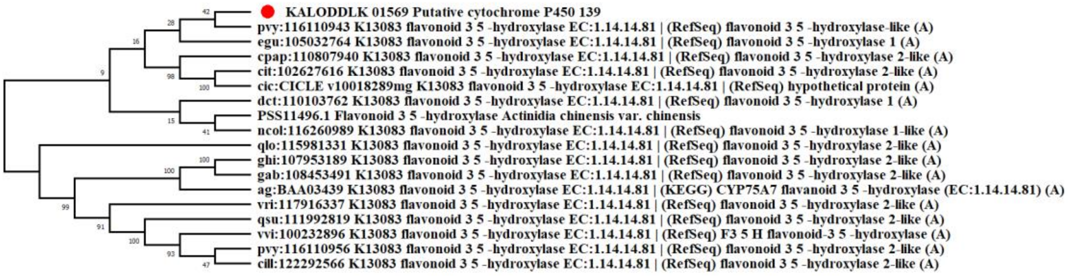


```
KALODDLK_01569      AVMKEVLRLHPILPLMVPHCPSESCVVGGYTIPKGARVFVNVWAIHRDPSIWENPTEFDP      60
PSS11496.1          AALKEVLRLYPPG-WMGSRRLSRDLIWGGVRLPRGALALYSPYLSGRDPAVWDRPDEFDP      59
                    *.:******:*     *   :   *..  :  **    :*:** .:  .  :    ***::*:.* ****

KALODDLK_01569      ERFVDGKGDYSGKDFTYFPFGSGRRICAGIAMAERMVMFSLASL        104
PSS11496.1          GRWAGKP-----PAWAYLPFGGGERLCLGMHLAQMLIHDALAAL        98
                     *:..          ::*:***.*.*:* *:  :*:  ::   :**:*
```

(7).DFR:

>KALODDLK_01230 dTDP-glucose 4,6-dehydratase

VLVTGASGFVGSWLVMKLLQAGYTVRATVRDPANVGKTKPLMDLP-GATERLSIW-KADLAEEGSFHDAIRGCTGVFHVATPMDFLSKDPENEVIKPTVEGMISIMRACKEAGTVRRIVFTSSAGTVNLEERQRPVYDEES-WTDVDFCRRVKMTGWMYFVSKTLAEKAALAYAAEHGLDLVTIIPTLVVGPFISASMPPSLITALA

>sp|P51108.1|DFRA_MAIZE RecName: Full=Dihydroflavonol 4-reductase; Short=DFR; AltName: Full=Dihydrokaempferol 4-reductase; AltName: Full=Flavanone 4-reductase; Short=FNR

VAVTGAEGFIGSHLVETLVRSGARVRAMVLYNSFGSWGWLDDLPPEILEH-VEVVLGDVRD-PVSVREFMRGAEVVYHLAALIAIPYSYAAPHSYVQTNVVGTLNVLEAARDLQTPRLV

HTSTSEVYGTARSVPITERHPLQGQSPYSATKI-AADKLVEAYHLSFGLPVVTLRPFNTYGPRQSARAVIPTIISQIA

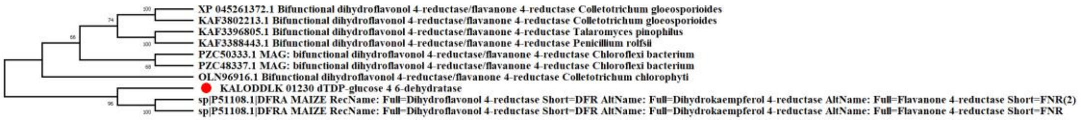


```
KALODDLK_01230          VLVTGASGFVGSWLVMKLLQAGYTVRATVRDPANVGKTKPLMDLPG-ATERLSIWKADLA      59
sp|P51108.1|DFRA_MAIZE  VAVTGAEGFIGSHLVETLVRSGARVRAMVLYNS-FGSWGWLDDLPPEILEHVEVVLGDVR      59
                        * ****.**:** ** .*:::*  *** *   : .*.   * ***    *::.:  .*:

KALODDLK_01230          EEGSFHDAIRGCTGVFHVATPMDFLSK-DPENEVIKPTVEGMISIMRACKEAGTVRRIVF      118
sp|P51108.1|DFRA_MAIZE  DPVSVREFMRGAEVVYHLAALIAIPYSYAAPHSYVQTNVVGTLNVLEAARDLQTPRL-VH      118
                        :  *.:: :**.   *:*:*: : :  .     :.  :: .* * :.::.*.::  * *  *.

KALODDLK_01230          TSSAGTVNLEERQRPVYDEESWTDVDFCRRVKMTGW-MYFVSKTLAEKAALAYAAEHGLD      177
sp|P51108.1|DFRA_MAIZE  TSTS-EVYGTARSVPI-----------TERHPLQGQSPYSATKIAADKLVEAYHLSFGLP      166
                        **::  *    *. *:              .*  : *   * .:*  *:* . **  ..**

KALODDLK_01230          LVTIIPTLVVGPFISAS-MPPSLITALA       204
sp|P51108.1|DFRA_MAIZE  VVTLRPFNTYGPRQSARAVIPTIISQIA       194
                        :**: *  . **  **  : *::*: :*
```

(8). FLS:

> KALODDLK_01423 2,3-dihydro-2,3-dihydroxybenzoate dehydrogenase

SSKVWLITGTSSGFGRSLVSSVLARGDRVIATSRSLEPIQHLKGTND-NLRLLQLDVTAGEEL-LQCKMKEAVSSWGRIDVLVNNAGSCHLGILEEGGSALLRRQYEVNVFGLLDVTNACLPHLRAQTEATIVVMGSRSAWTCENMGIGPYGSSKAAVHAVAETLSVEVAPFNIR-VLIVEPSAFRTRMVR

> KAF7344498.1 Flavonol synthase [Mycena sanguinolenta]

TARVALVTGAAQGIGAAVASALAARGARVLATDRQPIPPDLAAQPGVMPY-PLDVTDPRAAEQAVAHAEATLGPLTDLVNVAGVLRPGPLTDLSDEDWHATFAVNTSGVFFVSRAATRVMRARGRGSVVTVGSNAAHVPRTGMGAYASSKAAAAHL-TRTLGLELAGSGVRCNLVSPGSTDTPMQR

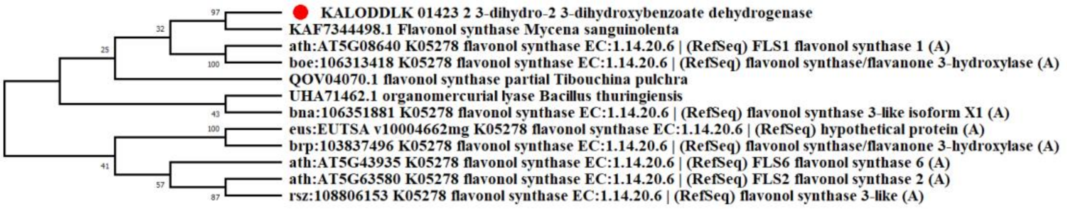


```
KALODDLK_01423     SSKVWLITGTSSGFGRSLVSSVLARGDRVIATSRSLEPIQHLKGTNDNLRLLQLDVTAGE      60
KAF7344498.1       TARVALVTGAAQGIGAAVASALAARGARVLATDRQPIPPDLAA--QPGVMPYPLDVTDPR      58
                   :::* *:**::.*:* ::.*:: *** **:**.*.   * :      : .:     ****  .

KALODDLK_01423     ELLQCKMKEAVSSWGRIDVLVNNAGSCHLGILEEGGSALLRRQYEVNVFGLLDVTNACLP      120
KAF7344498.1       AAE-QAVAHAEATLGPLTDLVNVAGVLRPGPLTDLSDEDWHATFAVNTSGVFFVSRAATR      117
                          : .* :: * :   *** **   : * * : ..    :   : **.  *:: *:.*.

KALODDLK_01423     HLRAQTEATIVVMGSRSAWTCENMGIGPYGSSKAAVHAVAETLSVEVAPFNIRVLIVEPS      180
KAF7344498.1       VMRARGRGSVVTVGSNAA-HVPRTGMGAYASSKAAAAHLTRTLGLELAGSGVRCNLVSPG      176
                    :**: ..::*.:**.:*      . *:* *.*****.   ::.**.:*:*  .:*  :*.*.

KALODDLK_01423     AFRTRMVR     188
KAF7344498.1       STDTPMQR     184
                   :  * * *
```

(9).ANS:

>KALODDLK_01230 dTDP-glucose 4,6-dehydratase

KTACVTGGSGYIGSALIKLLLEKGYAVKTTVRNPDDMEKNSHLKDLQKLG-PLTVFRADMDEEGSFDDAVAGCDYVFLVAAPLHFEAQDPEKEQIEPAIQGTLNTMRSCVKAGTVRRVILTSSVAAVYFRPDLLGDGHGHVLDEDSWSDVDFLRAHKPPT-WSHCVSKVLLEKEAGRFAEEHGISLVTILPVIVVGAAPAPKARSSIVDCLSMLS

> BAU68558.1 3-deoxyanthocyanidin synthase [Sorghum bicolor]

KLVAVTGAEGFIGSHLVETLVRSGARVRAMVLYNSFGSWGWLDDLPPEILEH-VEVVLGDVRD-PVSVREFMRGAEVVYHLAALIAIPYSYAAPHSYVQTNVVGTLNVLEAARDLQTPRLVHTSTSEVYGTARSVPITERHPLQGQSPYSATKI-AADKLVEAYHLSFGLPVVTLRPFNTYGPRQSARAVIPTIISQIA

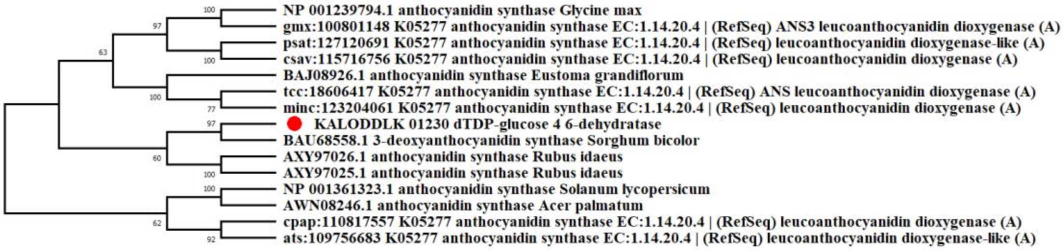


```
KALODDLK_01230    KTACVTGGSGYIGSALIKLLLEKGYAVKTTVRNPDDMEKNSHLKDLQ--KLGPLTVFRAD    58
BAU68558.1        KLVAVTGAEGFIGSHLVETLVRSGARVRAMVLYNS-FGSWGWLDDLPPEILEHVEVVLGD    59
                  * ..***..*:*** *:: *:..*  *:: *   .  :  .  . *.**    *  : *.  .*

KALODDLK_01230    MDEEGSFDDAVAGCDYVFLVA----APLHFEAQDPEKEQIEPAIQGTLNTMRSCVKAGTV    114
BAU68558.1        VRDPVSVREFMRGAEVVYHLAALIAIPYSYAAPH---SYVQTNVVGTLNVLEAARDLQTP    116
                  : :  *. : : *.: *: :*     *  : * .    . ::  : ****.:.:.  .  *

KALODDLK_01230    RRVILTSSVAAVYFRPDLLGDGHGHVLDEDSWSDVDFLRAHKPPTWSHCVSKVLLEKEAG    174
BAU68558.1        RLV--HTSTSEVYGTARSVPITERHPL-----------QGQSPY----SATKIAADKLVE    159
                  * *   :*.: **     :    . * *            :.:.*     ..:*:   :* .

KALODDLK_01230    RFAEEHGISLVTILPVIVVGAAPAPKARSSIVDCLSMLS    213
BAU68558.1        AYHLSFGLPVVTLRPFNTYGPRQS--ARAVIPTIISQIA    196
                   :  ..*: :**: *. . *   :  **: *   :* ::
```

(10).BZ1:

> KALODDLK_00889 O-mycaminosyltylonolide 6-deoxyallosyltransferase

QVELFVTHGGLNSTYEGLAAGKPLVVMPFFGDQPVNAQHIVNKGL-GAQVDPWTLTAAKLTRAIEAQLADREAA

> XP_013906137.1 Anthocyanidin 3-O-glucosyltransferase [Monoraphidium neglectum]

RVAAAVHHGGAGTTAAGLAAGVPNVVVPFFGDQPFWGDRV-QRLGVGPAPVPRRALNERTLADALTRAVTDAGMRDRAAA

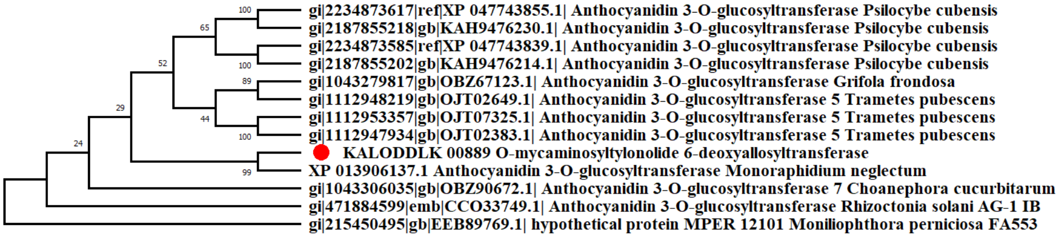


```
KALODDLK_00889    QVELFVTHGGLNSTYEGLAAGKPLVVMPFFGDQPVNAQHIVNKGLGAQVDPWTLTA-AKL    59
XP_013906137.1   RVAAAVHHGGAGTTAAGLAAGVPNVVVPFFGDQPFWGDRVQRLGVGPAPVPRRALNERTL    60
                 :*    * *** .:*   ***** * **:*******. .::: . *:*     *       .*

KALODDLK_00889    TRAI-----EAQLADREAA 73
XP_013906137.1   ADALTRAVTDAGMRDRAAA 79
                 : *:      :* : ** **
```

(11).F3’M:

>KALODDLK_01569 Putative cytochrome P450 139

AVMKESLRLHPPAPLLVPHCPSEDSLLGGFRVPKGSRVFVNVWAIHRDPT-LWENPTEFDP-DRFVNGGVDYSGNDFSYLPFGSGRRMCAGMAMAERMVMYSLTTLLHSFNWELPKGEKLDLSEQFGIVLKKKLPLI

> XP_031376784.1 flavonoid 3'-monooxygenase CYP75B137-like [Punica granatum]

AALKEVLRLYPPGWMGSRRLSRDLIWGGVRLPRGALALYSPYLSGRD-PAVWDRPDEFDPGRWAGKPPAWAYLPFGGGERLCLGMHLAQMLIHDALAALPPLHAVRGDATPLPGLTLGPRGPLI

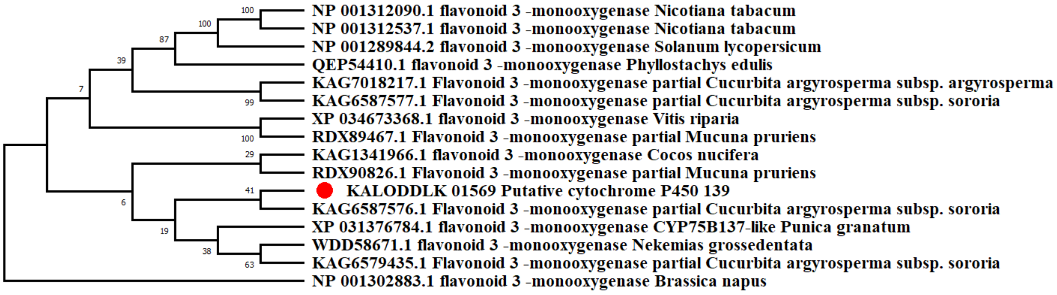


```
KALODDLK_01569      AVMKESLRLHPPAPLLVPHCPSEDSLLGGFRVPKGSRVFVNVWAIHRDPTLWENPTEFDP      60
XP_031376784.1      AALKEVLRLYPPGWM-GSRRLSRDLIWGGVRLPRGALALYSPYLSGRDPAVWDRPDEFDP      59
                    *.:** ***:**.  :    :   *.* : **.*:*:*: .: .  :   ***::*:.* ****

KALODDLK_01569      DRFVNGGVDYSGNDFSYLPFGSGRRMCAGMAMAERMVMYSLTTLLHSFNWELPKGEKLDL      120
XP_031376784.1      GRWAGK-----PPAWAYLPFGGGERLCLGMHLAQMLIHDALAALPPLHA---VRG---DA      108
                    .*:.        ::*****.*.*:* ** :*: ::  :*::*    .      :*   *

KALODDLK_01569      SEQFGIVLKKKLPLI      135
XP_031376784.1      TPLPGLTLGPRGPLI      123
                    :    *:.*   : ***
```

(12).R06611(Flavonol 3-O-glucosyltransferase):

>KALODDLK_00889 O-mycaminosyltylonolide 6-deoxyallosyltransferase

PAEEVVAWLDACPPRSVVYVCFGSQYTPTEKQGRA-LAAALERSGVRFVWAIGGGGAV-VPEGFEGRVVEILNHVAVGAFVTHCGWNSVLEAVAAGVVLLAWPMRADQFANARLVVEELGVAVRVGEGEEGTPEPEDLARVMAEAVAEVAWPEMRVRAAALGRK

>XP_029116545.1 flavonol 3-O-glucosyltransferase UGT89B1-like [Elaeis guineensis]

PPPALEAFLNSGPPPVSIGFGSMTTPDPQVTTRAVVAALARSGQRAVLLSGWG-GLSAADVPDTVFVTDSVPHDWLFPRVAAAVHHGGAGTTAAGLAAGVPNVVVPFFGDQPFWGDRVQRLGVGPAPVPRRALNERTLADALTRAVTDAGMRDRAAALGAR

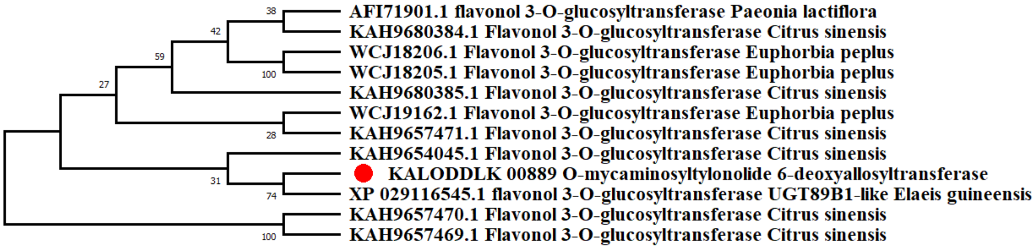

```
KALODDLK_00889      PAEEVVAWLDACPPRSVVYVCFGSQYTPTE-KQGRALAAALERSGVRFVWAIGGGGAVVP      59
XP_029116545.1      PPPALEAFLNSGPPP--VSIGFGSMTTPDPQVTTRAVVAALARSGQRAVLLSGWGGLSAA      58
                    *   : *:*:: **   * : ***  **      **:.*** *** * *   * **  .

KALODDLK_00889      EGFEGRVV-EILN----HVAVGAFVTHCGWNSVLEAVAAGVVLLAWPMRADQ-FANARLV      113
XP_029116545.1      DVPDTVFVTDSVPHDWLFPRVAAAVHHGGAGTTAAGLAAGVPNVVVPFFGDQPFWGDRV-      117
                    :  :  .* : :     .   *.* * * * .:.   .:****  :.  *: .** * .  *:

KALODDLK_00889      VEELGVAVRVGEGEEGTPEPEDLARVMAEAV-AEVAWPEMRVRAAALGRK  162
XP_029116545.1      -------QRLGVGPAPVPRRALNERTLADALTRAVTDAGMRDRAAALGAR  160
                           *:* *   .*.      *.:*:*:   *:    ** ****** :
```

(13).UGT75C1(Cyanidin 3-O-glucoside 5-O-glucosyltransferase):.

>KALODDLK_02100 Beta-glucosidase.

DRLDFPKHFIFGASSCAYQVEGAAFEDGRTLSTFDIAAHSGHLPGNG-
DITSDEYHKYKEDVELMVETGLDAYRFSISWSRLIPNGRGPVNPKGLEYYNNLVNALL
TKGTQPHVTLLHSDLPQALRDEYGGLFISPKFIDDFVAYADVCFREFGDRVLHWTT-
FNE-
ANFLAFGDENTPASALYLSAHHLLLAHASATRLYRENYQASQRGFIGINVYAYDFIPET
NTEVDVIAAKRARDFFIGWFVQPLMNGEYPLTMRKNGGPRLPKFTPNETELLTGSYD-
FIGL-
NYYTAKTVKDDPVMLTVEPRNYYTDQGLISSYLGNIDPYQGHPFFNTPWGLHDVLQQ
FKQVYGNPPVYIHENGEVGDHDADYDKLINDIPRVEYLQGHIRAVLDA-
VRNGSNVKGYFVWSFLDMYELMYGTKFTFGLYYIDFNDPKLTRHPKLSQKWYSRFL.

>sp|E3W9M2.1|AA5GT_DIACA RecName: Full=Cyanidin 3-O-glucoside 5-O-glucosyltransferase (acyl-glucose).

DPARFPARFTWGVATSSYQIEGAPREDGKGPSIWDTFCRTPGKVRGGDTGDVAC-
DH-
YHRLDSDLDMIRDLGVNAYRFSISWPRILPHGRGAVNQAGLDFYQRLVDGLLTRGITP
WATLYHWDLPQTLEDEGGWTVRGTAEAFGTYSAVVAAALGDRVKHFITLNEP-
WCSAYLGYGI-
GIHAPGRHDLRASFAATHHLLVGHGRAMQAIRGQAPGAQAGITLNLHHTYPATDTP
ADRAAAYRMDGFQNRWYLDPVYGRGYPQDMVDLLGDL-
SPQAQGLVLPGDTELMGQPTD-
FLGVNMYSRAVGQDAPGEGFLHARQIRPEGSAYTGFDWEVAPDSLTDLLVRLQEDYA
PDAIYITENGSTYPDVADEDGNVNDLERTQYLTEHLAATQEALARGAKVAGY-
FAWSLMDNFEWAEGYDKRFGIVHVDFDTQVRTPKLSGRTYRDFL.

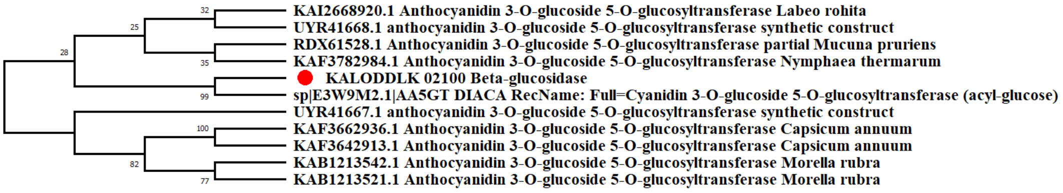

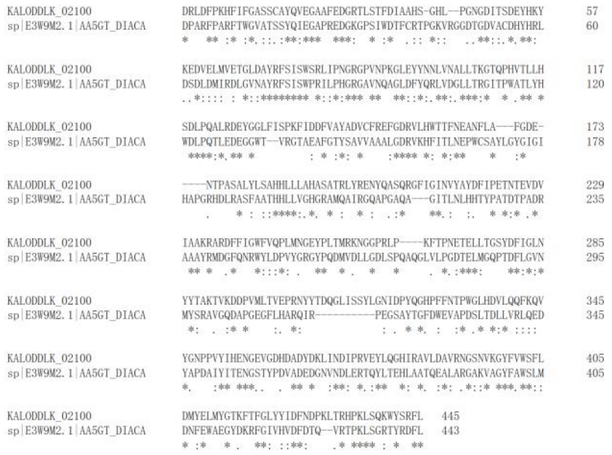

(14). AA5GT:

>KALODDLK_02100 Beta-glucosidase

DRLDFPKHFIFGASSCAYQVEGAAFEDGRTLSTFDIAAHSGHLPGNG-
DITSDEYHKYKEDVELMVETGLDAYRFSISWSRLIPNGRGPVNPKGLEYYNNLVNALL
TKGTQPHVTLLHSDLPQALRDEYGGLFISPKFIDDFVAYADVCFREFGDRVLHWTT-
FNE-
ANFLAFGDENTPASALYLSAHHLLLAHASATRLYRENYQASQRGFIGINVYAYDFIPET
NTEVDVIAAKRARDFFIGWFVQPLMNGEYPLTMRKNGGPRLPKFTPNETELLTGSYD-
FIGL-
NYYTAKTVKDDPVMLTVEPRNYYTDQGLISSYLGNIDPYQGHPFFNTPWGLHDVLQQ
FKQVYGNPPVYIHENGEVGDHDADYDKLINDIPRVEYLQGHIRAVLDA-
VRNGSNVKGYFVWSFLDMYELMYGTKFTFGLYYIDFNDPKLTRHPKLSQKWYSRFL

>sp|E3W9M2.1|AA5GT_DIACA RecName: Full=Cyanidin 3-O-glucoside 5-O-glucosyltransferase (acyl-glucose)

DPARFPARFTWGVATSSYQIEGAPREDGKGPSIWDTFCRTPGKVRGGDTGDVAC-
DH-
YHRLDSDLDMIRDLGVNAYRFSISWPRILPHGRGAVNQAGLDFYQRLVDGLLTRGITP
WATLYHWDLPQTLEDEGGWTVRGTAEAFGTYSAVVAAALGDRVKHFITLNEP-
WCSAYLGYGI-
GIHAPGRHDLRASFAATHHLLVGHGRAMQAIRGQAPGAQAGITLNLHHTYPATDTP
ADRAAAYRMDGFQNRWYLDPVYGRGYPQDMVDLLGDL-
SPQAQGLVLPGDTELMGQPTD-
FLGVNMYSRAVGQDAPGEGFLHARQIRPEGSAYTGFDWEVAPDSLTDLLVRLQEDYA
PDAIYITENGSTYPDVADEDGNVNDLERTQYLTEHLAATQEALARGAKVAGY-
FAWSLMDNFEWAEGYDKRFGIVHVDFDTQVRTPKLSGRTYRDFL

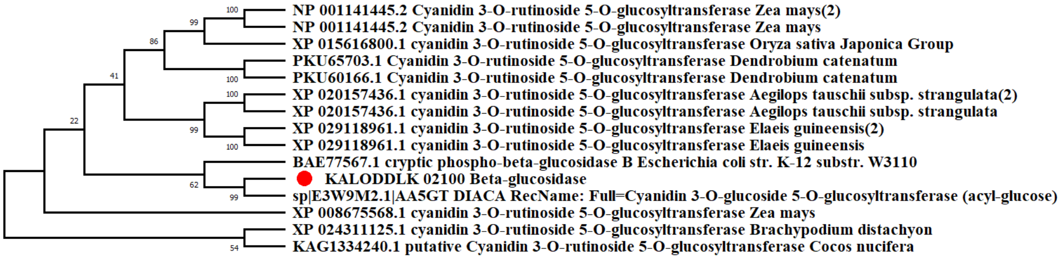

```
KALODDLK_02100              DRLDFPKHFIFGASSCAYQVEGAAFEDGRTLSTFDIAAHS-GHL--PGNGDITSDEYHKY      57
sp|E3W9M2.1|AA5GT_DIACA     DPARFPARFTWGVATSSYQIEGAPREDGKGPSIWDTFCRTPGKVRGGDTGDVACDHYHRL      60
                            *   ** :* :*.::.:**:***  ***:  * :*  .:: *::   ..**::.*.**:

KALODDLK_02100              KEDVELMVETGLDAYRFSISWSRLIPNGRGPVNPKGLEYYNNLVNALLTKGTQPHVTLLH      117
sp|E3W9M2.1|AA5GT_DIACA     DSDLDMIRDLGVNAYRFSISWPRILPHGRGAVNQAGLDFYQRLVDGLLTRGITPWATLYH      120
                            ..*:::: : *::********* *::*:*** **  **::*:.**:.***:*  * .** *

KALODDLK_02100              SDLPQALRDEYGGLFISPKFIDDFVAYADVCFREFGDRVLHWTTFNEANFLA---FGDE-      173
sp|E3W9M2.1|AA5GT_DIACA     WDLPQTLEDEGGWT--VRGTAEAFGTYSAVVAAALGDRVKHFITLNEPWCSAYLGYGIGI      178
                             ****:*.** *         : * :*: *    :**** *: *:**    *   :*

KALODDLK_02100              ----NTPASALYLSAHHLLLAHASATRLYRENYQASQRGFIGINVYAYDFIPETNTEVDV      229
sp|E3W9M2.1|AA5GT_DIACA     HAPGRHDLRASFAATHHLLVGHGRAMQAIRGQAPGAQA---GITLNLHHTYPATDTPADR      235
                                .    * : ::****:.*. * :  * :  .:*    **.:  :.  * *:* .*

KALODDLK_02100              IAAKRARDFFIGWFVQPLMNGEYPLTMRKNGGPRLP----KFTPNETELLTGSYDFIGLN      285
sp|E3W9M2.1|AA5GT_DIACA     AAAYRMDGFQNRWYLDPVYGRGYPQDMVDLLGDLSPQAQGLVLPGDTELMGQPTDFLGVN      295
                             ** *  .*   *:::*: .   **  * .  *   *     . *.:***:    **:*:*

KALODDLK_02100              YYTAKTVKDDPVMLTVEPRNYYTDQGLISSYLGNIDPYQGHPFFNTPWGLHDVLQQFKQV      345
sp|E3W9M2.1|AA5GT_DIACA     MYSRAVGQDAPGEGFLHARQIR----------PEGSAYTGFDWEVAPDSLTDLLVRLQED      345
                             *:  . :* *    :. *:                : .  * *. :  :* .* *:* ::::

KALODDLK_02100              YGNPPVYIHENGEVGDHDADYDKLINDIPRVEYLQGHIRAVLDAVRNGSNVKGYFVWSFL      405
sp|E3W9M2.1|AA5GT_DIACA     YAPDAIYITENGSTYPDVADEDGNVNDLERTQYLTEHLAATQEALARGAKVAGYFAWSLM      405
                            *.    :** ***..   . ** *  :**: *.:**  *: *.  :*: .*::* ***.**::

KALODDLK_02100              DMYELMYGTKFTFGLYYIDFNDPKLTRHPKLSQKWYSRFL    445
sp|E3W9M2.1|AA5GT_DIACA     DNFEWAEGYDKRFGIVHVDFDTQ--VRTPKLSGRTYRDFL    443
                            * :*   * .   **: ::**:    .* **** : *  **
```

HPLC results

In Figure S2c, Eluting peaks of calibrator were observed at 4.4min, 5.9min and 6.2min, represent quercetin, kaempferol and isopurine respectively. The production of flavonoid during fermentation is determined by its peak area (mAU) and its properties can be determined by the time of peaking. **Figure S2a** shows the content of each substance in the blank medium, three flavonoids were not detected**. Figure S2b** represents bacterial fermentation broth**,** quercetin was identified**.**

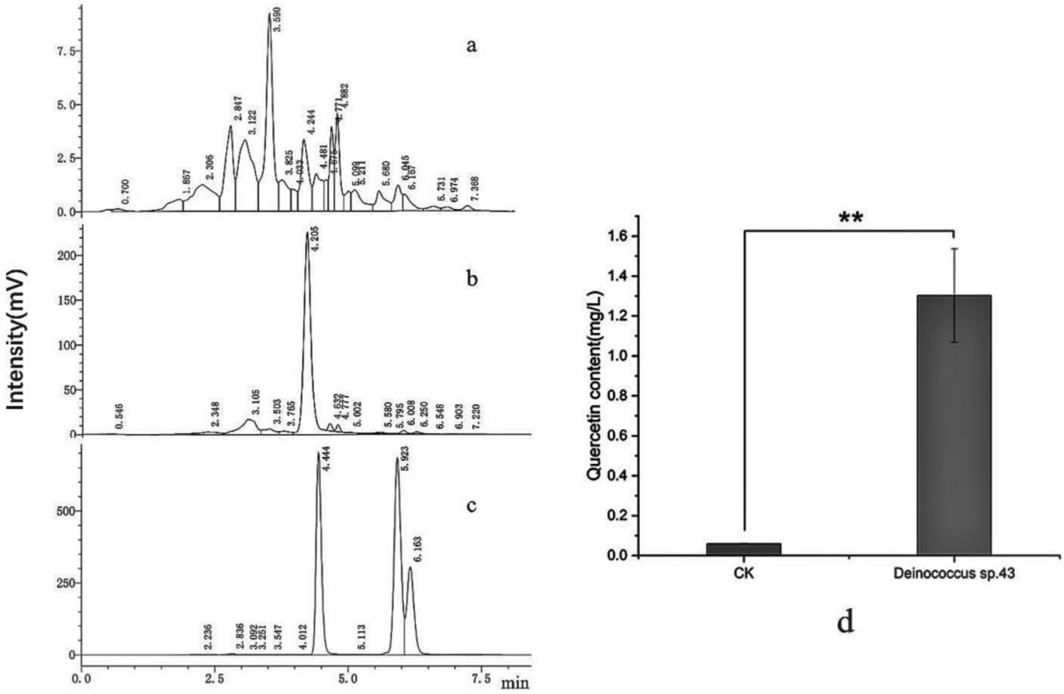

Figure S2. In FS2(c), Eluting peaks of calibrator were observed at 4.4min, 5.9min and 6.2min, represent quercetin, kaempferol and isopurine respectively. The production of flavonoid during fermentation is determined by its peak area (mAU) and its properties can be determined by the time of peaking. FS2(a) shows the content of each substance in the blank medium, three flavonoids were not detected. FS2(b) represents bacterial fermentation broth, quercetin was identified.

LC-MS detection quality control.

The repeatability of metabolite extraction and instrument detection can be judged by analyzing the overlap of mass spectrometry total ion chromatograms (TIC diagram) of three QC samples. **Figure S3** shown the TIC of three QC samples in positive ion and negative ion modes, which reflected the reliability of sample preparation process and LC-MS detection.

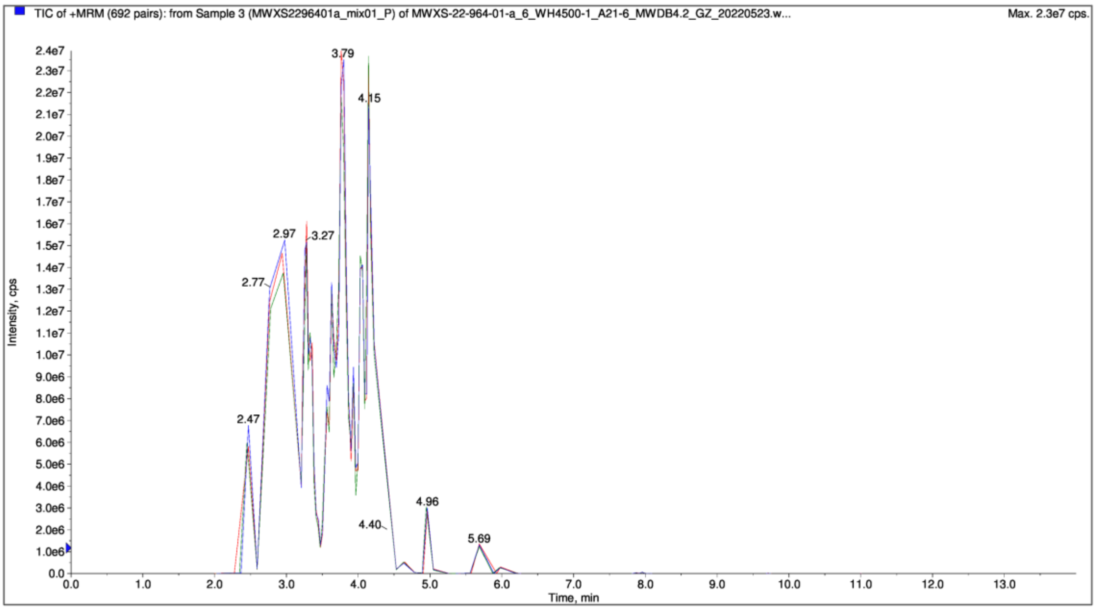

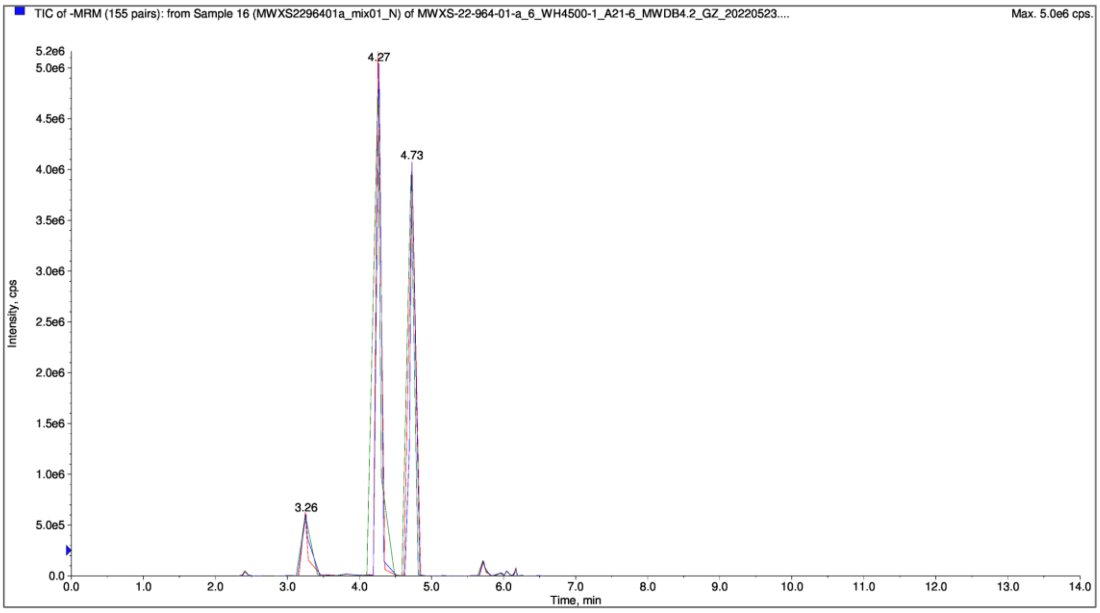

Figure S3. The repeatability of metabolite extraction and instrument detection can be judged by analyzing the overlap of mass spectrometry total ion chromatograms (TIC diagram) of three QC samples. FS3 shown the TIC of three QC samples in positive ion and negative ion modes, which reflected the reliability of sample preparation process and LC-MS detection.

Based on metabolite database analysis, we performed qualitative analysis of all detected ion types. In the multi-peak diagram of MRM metabolite detection in multi reaction monitoring mode, the samples that can be detected in positive ion and negative ion modes are displayed, and the peaks of different colors represent different metabolites (**Figure S4**). After filtering the blank control signal, there were relatively many kinds of

metabolites in the sample, and the number of peaks with strong signal was moderate, which was an ideal microbial experimental model.

positive ion modes

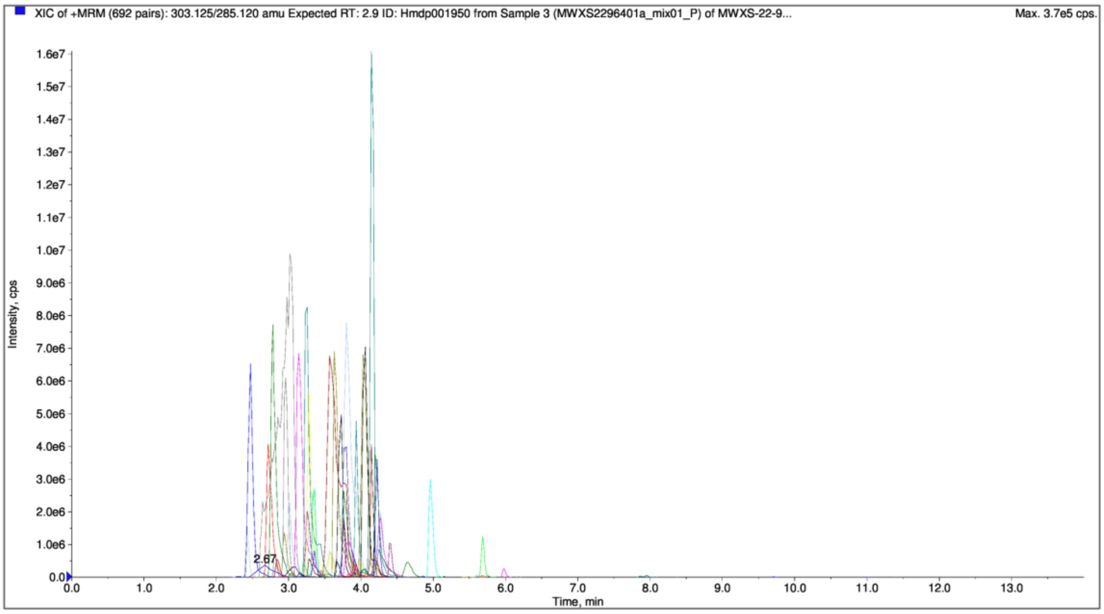

negative ion modes

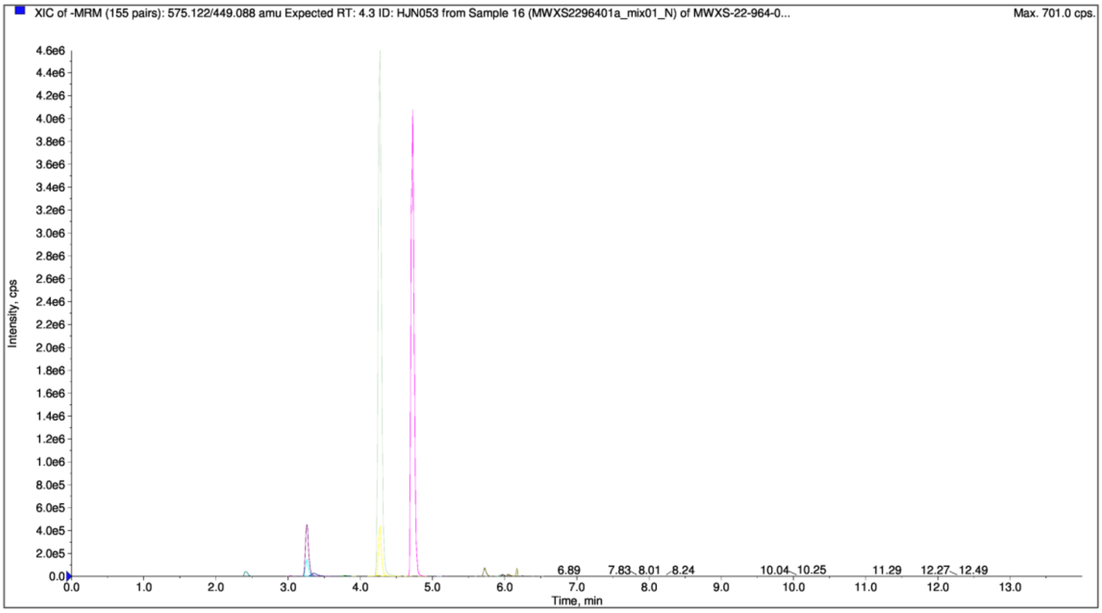

Figure S4. Based on metabolite database analysis, we performed qualitative analysis of all detected ion types. In the multi-peak diagram of MRM metabolite detection in multi reaction monitoring mode, the samples that can be detected in positive ion and negative ion modes are displayed, and the peaks of different colors represent different metabolites (FS4). After filtering the blank control signal, there were relatively many kinds of metabolites in the sample, and the number of peaks with strong signal was moderate, which was an ideal microbial experimental model.
